# Comparison common equations for LDL-C calculation with direct assay and developing a novel formula in Iranian children and adolescents: the CASPIAN V study

**DOI:** 10.1186/s12944-020-01306-7

**Published:** 2020-06-06

**Authors:** Fatemeh Molavi, Nazli Namazi, Mojgan Asadi, Mahnaz Sanjari, Mohammad Esmaeil Motlagh, Gita Shafiee, Mostafa Qorbani, Ramin Heshmat, Roya Kelishadi

**Affiliations:** 1 Chronic Diseases Research Center, Endocrinology and Metabolism Population Sciences Institute, Tehran University of Medical Sciences, Tehran, Iran; 2 Diabetes Research Center, Endocrinology and Metabolism Clinical Sciences Institute, Tehran University of Medical Sciences, Tehran, Iran; 3 Department of Pediatrics, Ahvaz Jundishapur University of Medical Sciences, Ahvaz, Iran; 4 Non-communicable Diseases Research Center, Alborz University of Medical Sciences, Karaj, Iran; 5 Endocrinology and Metabolism Research Center, Endocrinology and Metabolism Clinical Sciences Institute, Tehran University of Medical Sciences, Tehran, Iran; 6 Child Growth and Development Research Center, Research Institute for Primordial Prevention of Non-communicable Disease, Isfahan University of Medical Sciences, Isfahan, Iran

**Keywords:** LDL level, Triglyceride, Children and adolescent

## Abstract

**Background:**

Hypercholesterolemia is a common dyslipidemia that leads to atherosclerosis. It is proved that early stages of atherosclerosis begins in early stages of life. In several studies, widespread prevalence of dyslipidemia in children is reported. So, assessment of lipid profile in children and adolescence is necessary for early diagnosis of dyslipidemia. Laboratory methods for measuring LDL are not available and economical. So, in some laboratories Friedwald method is used to determine LDL level. But, the preciseness of this method is not acceptable. Further, the preciseness of this method was not assayed in children and adolescence. So, it seems that assaying the preciseness of different methods is necessary.

**Methods:**

The methodology of this work is on the basis of findings of the Caspian V study. This study was conducted in 30 provinces of Iran during 2015. The population of this work was rural and urban students aged 7–18 years old. The level of total cholesterol (TC), HDL, LDL, and TG were measured using laboratory methods. The average and variances values were determined for each group of data using SPSS. Further, LDL values were calculated with a new formula introduced in this work. A comparison was made between the new formula and the other methods.

**Results:**

In the present study, we found that compare to four common formulas, Friedwald was the best equation to estimate LDL-C concentrations in Iranian children and adolescents and the new formula was the next accurate equation. The strongest correlation between Friedwald and the new equation was found for those with 15–18 years old.

**Conclusion:**

Considering the cut-off points of TG (100 mg/dL), we observed the strongest correlation between Friedwald equation and direct assay and the weakest one was for Ahmadi formula in subjects with either greater or lower TG concentrations. Furthermore, we found that Anandraja equation had the most sensitivity (89.5%), while the most specificity was dedicated to the new formula (98.9%).

## Introduction

According to pediatric advisory groups, selective screening for dyslipidemia in children is recommended particularly in those aged 2–18 years old with parents with precholestrolemia or other risk factors such as obesity and smoking. As there is an association between hyperlipidemia and cardiovascular diseases, controlling lipid profile can be helpful for primary and secondary preventions [1]. Since the basis of atherosclerosis and cardiovascular diseases start at early childhood, paying attention to lipid status is appreciated [2].

One of the crucial parameters used for CVD risk assessment is the serum level of low density lipoprotein-Cholesterol (LDL-C) [3, 4]. Various methods are used for measuring LDL-C concentrations. Although gold standard for LDL-C measurement is Ultracentrifugation following by betaquantification [5], it has several limitations. It is an expensive and time-consuming method that needs special equipments [6]. Therefore, it is not a common method for routine clinical measurements. Instead, other direct methods including homogenous assay techniques are usually used as well as various equations such as Friedewald, Chen and Anandaraja [7].

Although Friedewald formula is wildly used for reporting LDL-C, it features a fixed triglyceride (TG): very-low-density lipoprotein cholesterol (VLDL-C) ratio of 5:1. Accordingly, it cannot show the substantial inter-individual variability in TG: VLDL-C ratios [8]. Besides, the Friedewald equation is not applicable for those with fasting TG equal or higher than 400 mg/dL, and often this equation underestimates LDL-C concentrations in subjects with TG equal to 150 mg/dL. Another limitation of Friedwald formula is related to being 8 h overnight fasting that is usually difficult for children. Finding non-fasting measurement method with acceptable accuracy for LDL-C is practically preferred in children [9].

Several studies compared the amount of LDL-C concentration obtained from formulas with each other or with a direct measurement [6, 8–13]. However, it seems most studies examined adult populations. To the best of our knowledge, there is few studies in which common equations for LDL-C calculation were compared with direct assay in children and adolescents at national level.

## Material & Methods

### Study population and sampling framework

The present cross-sectional study was conducted on a sub sample from the CASPIAN V study, a population-based study in Iran, on students aged 7 to 18 years old. To choose eligible individuals, multistage, cluster sampling method was used from 30 provinces in 2015. Details of sampling procedures was presented elsewhere [14]. Briefly, in each province, children and adolescent considering equal number for boys and girls stratified based on living place (urban/ rural) as well as the level of education (primary /secondary). To reach the calculated number of participants, multistage, stratified cluster sampling method was also applied in each province. The size of cluster was 10 (10 students with their parents). Of 14,400 students in the CASPIAN study, 3844 students were selected for biochemical measurements. It means 14 out of 48 clusters from each province were randomly selected for the current study.

In the first step, for eligible students and their parents, sufficient explanations regarding the purpose of the study and the procedures were provided. Then, written informed consent and verbal consent were obtained from parents and students, respectively. All assessments were performed for subjects who completed the written informed consent.

Health-care professional team asked characteristics of participants and completed all questionnaires at schools in a room, where away from busy classrooms and interviewing with at least one of students’ parents.

### Biochemical assessments

Eligible students with at least one of their parents were referred to the laboratory for biochemical tests. After 12 h overnight fasting, 6 mL venous blood sample was collected from students. All blood samples were centrifuged at 2500–3000×g for 10 min and then serum samples were aliquot and stored at − 70 °C till measurement. Lipid profiles including TG, total cholesterol (TC), LDL-C and high-density lipoprotein-cholesterol (HDL-C) were measured using enzymatic method by Hitachi Auto Analyzer (Tokyo, Japan).

### LDL-C calculation

Apart from the measurement of LDL-C in serum samples, the amount of LDL-C was calculated using 4 common formulas as represented in Table 1.

**Table 1:** Different equations for LDL-C calculation

Researchers	Formulas
Friedwald	LDL = TC-HDL-(TG/5)
Ahmadi et al.	LDL = TC/1.19 + TG/1.9-HDL/1.1–38
Chen	LDL = (0.9 × TC)-(0.9× HDL)-(0.1 × TG)
Anandaraja	LDL = (0.9 × TC)-(0.9 × TG/5)-28
New formula	LDL = (0.97 × TC)-(0.93 × HDL)-(0.19 × TG)

Statistical analysis was performed on the data obtained from this assessed population. Accordingly, a regression model was developed. The developed model is as follows:

1 $$ {x}_{LDL-C}=\left( 0.97\times {x}_{TC}\right)-\left( 0.19\times {x}_{TG}\right)-\left( 0.93\times {x}_{HDL-C}\right) $$

where x_LDL-C_, x_TC_, x_TG_, and x_HDL-C_ are values of LDL-C, TC, TG, and HDL-C, respectively.

It is worthwhile noting that this developed model was obtained from the subjects with TG < 100 mg/dL. Note that this regression model was extracted from data using SPSS software (SPSS Inc., USA). The mentioned equation was then examined and validated on children and adolescents with TG > 100 mg/dL.

### Statistical analysis

The correlation among equations and with a direct measurement was examined. Findings were reported in subjects with TG > 100 mg/dL and < 100 mg/dL, separately.

The coefficient of determination is an index for assessing the correlation between actual and predicted values. This index is calculated as follows:

$$ {\mathrm{R}}^2=1-\frac{\sum {\left({\mathrm{x}}_{\mathrm{act}.}-{\mathrm{x}}_{\mathrm{pred}.}\right)}^2}{\sum {\left({\mathrm{x}}_{\mathrm{act}.}-{\mathrm{x}}_{\mathrm{ave}.}\right)}^2} $$

$$ {\mathrm{x}}_{\mathrm{ave}.}=\frac{\sum {\mathrm{x}}_{\mathrm{act}.}}{\mathrm{n}} $$

where x_act._, x_pred._, and n are the actual value, predicted value, and number of data, respectively.

Considering 110 mg/dL as a cut-off, sensitivity and specificity for each formula in all participants as well as those with low (< 100 mg/dL) and high TG (> 100 mg/dL) were presented. Youden index, (sensitivity + specificity)-100, was calculated for each in order to identify the best formula for LDL-C calculation in Iranian children and adolescents.

## Results

Findings are presented for 3844 children and adolescents categorized based on gender, age and residential place. As depicted in Table 2, the frequency of subjects with TG > 100 mg/dL was greater than those with TG < 100 mg/dL in all age categories (higher than 70% for all). The percentage of boys with TG > 100 mg/dL was higher than girls (73.1 vs.71.4%). However, the difference between genders was not considerable. Classifications by residential place showed that participants lived in rural places had higher TG concentrations than whom resident in urban regions (73.5 vs.71.8%, respectively).

**Table 2:** Frequency of subjects with TG levels lower and higher than 100 mg/dl in terms of age, sex and region

		Total		TG > 100		TG < 100	
		N	Percentage	N	Percentage	N	Percentage
Age	7–10	1147	29	848	73.9	299	26.1
	11–14	1655	44	1198	72.4	457	27.6
	15–18	1042	27	733	70.3	309	29.7
Sex	Female	1831	47.6	1307	71.4	524	28.6
	Male	2013	52.4	1472	73.1	541	26.9
Region	Urban	2749	72	1994	71.8	782	28.2
	Rural	1068	28	785	73.5	283	26.5

Based on Table 3, there were no significant differences in LDL-C concentrations obtained from formulas except Anandraja (*p* = 0.18) when subjects were classified based on the cut-off point of 100 mg/dL for TG.

**Table 3:** Mean and standard deviation of lipid levels

	Total		TG > 100		TG < 100		*P*-value
	Average	SD	Average	SD	Average	SD	
Cholesterol	153.8	27.4	149.99	25.60	163.91	29.39	< 0.001
HDL	46.2	9.9	47.58	10.07	42.55	8.17	< 0.001
Direct LDL	90.29	22.64	89.31	21.65	92.86	24.87	< 0.001
Friedwald LDL	90.17	22.21	89.16	21.13	92.80	24.87	< 0.001
Ahmadi LDL	95.80	38.06	80.30	22.61	136.23	40.43	< 0.001
Chen LDL	88.09	21.03	85.46	19.80	94.94	22.54	< 0.001
Anandraja LDL	94.61	23.39	92.94	22.63	93.81	25.26	0.188
New formula	89.55	22.01	88.50	21.13	92.28	24.62	< 0.001

In Table 4, the correlation between predicted formulas with each other and direct assay are provided. In general, Friedwald formula (*r* = 0.982) stood at the first rank for the correlation with direct assay and the second rank was dedicated to the new formula (*r* = 0.978). The lowest correlation was observed for Ahmadi formula (*r* = 0.553). In subjects with TG > 100 mg/dL, the strongest correlation was found between direct assay and Friedwald equation (*r* = 0.979) followed by the new formula (*n* = 0.974). The weakest correlation was observed for Ahmadi formula (*r* = 0.839). Similar findings were obtained for those with TG > 100 mg/dL. However, stronger correlation was obtained for Friedwald (*r* = 0.986) and lowest association was seen for Ahmadi formula (*r* = 0.553) compared to whom with TG < 100 mg/dL.

**Table 4:** Correlatin between LDL-C level for different methods

		Ahmadi LDL	Friedwald LDL	Direct LDL	Anandraja LDL	Chen LDL	New formula
TG < 100	Ahmadi LDL	1	0.857	0.839	0.729	0.880	0.847
	Friedwald LDL	0.857	1	0.979	0.913	0.989	0.991
	Direct LDL	0.839	0.979	1	0.901	0.972	0.974
	Anandraja LDL	0.729	0.913	0.901	1	0.910	0.925
	Chen LDL	0.880	0.989	0.972	0.910	1	0.998
	New formula	0.847	0.991	0.974	0.925	0.998	1
TG > 100	Ahmadi LDL	1	0.515	0.503	0.445	0.658	0.520
	Friedwald LDL	0.515	1	0.986	0.954	0.984	1.00
	Direct LDL	0.503	0.986	1	0.942	0.969	0.986
	Anandraja LDL	0.445	0.954	0.942	1	0.930	0.957
	Chen LDL	0.658	0.984	0.969	0.930	1	0.985
	New formula	0.520	1.00	0.986	0.957	0.985	1
Total	Ahmadi LDL	1	0.566	0.553	0.430	0.696	0.566
	Friedwald LDL	0.566	1	0.982	0.922	0.979	0.994
	Direct LDL	0.553	0.982	1	0.910	0.963	0.978
	Anandraja LDL	0.430	0.922	0.910	1	0.893	0.931
	Chen LDL	0.696	0.979	0.963	0.893	1	0.986
	New formula	0.566	0.994	0.978	0.931	0.986	1

As presented in Table 5, the strongest correlation between Friedwald and the new equation was found for those with 15–18 years old (*r* = 0.987), while the weakest correlation was related to Ahmadi formula for whom with 11–14 years old (*r* = 0.545).

**Table 5:** Correlation between different LDL-C calcuation methods in terms of age

			Ahmadi LDL	Friedwald LDL	Direct LDL	TG	TC	HDL	Anandraja LDL	Chen LDL	New formula
Age	7–10	Ahmadi LDL	1	0.576	0.562	0.865	0.691	−0.145	0.440	0.699	0.576
		Friedwald LDL	0.576	1	0.977	0.090	0.913	0.176	0.924	0.980	0.994
		Direct LDL	0.562	0.977	1	0.090	0.913	0.176	0.924	0.980	0.974
		TG	0.865	0.090	0.088	1	0.291	−0.270	−0.022	0.254	0.095
		TC	0.691	0.913	0.896	0.291	1	0.420	0.950	0.938	0.923
		HDL	−0.145	0.176	0.179	−0.270	0.420	1	0.527	0.122	0.186
		Anandraja LDL	0.440	0.924	0.908	−0.022	0.950	0.527	1	0.897	0.933
		Chen LDL	0.699	0.980	0.960	0.254	0.938	0.122	0.897	1	0.987
		New formula	0.576	0.994	0.974	0.095	0.923	0.186	0.933	0.987	1
	11–14	Ahmadi LDL	1	0.559	0.545	0.884	0.691	−0.173	0.416	0.691	0.557
		Friedwald LDL	0.559	1	0.985	0.107	0.901	0.131	0.917	0.975	0.991
		Direct LDL	0.545	0.985	1	0.099	0.891	0.133	0.908	0.963	0.980
		TG	0.884	0.107	0.099	1	0.329	−0.267	−0.009	0.282	0.113
		TC	0.691	0.901	0.891	0.329	1	0.380	0.941	0.933	0.914
		HDL	−0.173	0.131	0.133	−0.267	0.380	1	0.498	0.070	0.137
		Anandraja LDL	0.416	0.917	0.908	−0.009	0.941	0.498	1	0.887	0.927
		Chen LDL	0.691	0.975	0.963	0.282	0.933	0.070	0.887	1	0.985
		New formula	0.557	0.991	0.980	0.113	0.914	0.137	0.927	0.985	1
	15–18	Ahmadi LDL	1	0.568	0.557	0.879	0.714	−0.146	0.443	0.704	0.571
		Friedwald LDL	0.568	1	0.987	0.107	0.909	0.141	0.929	0.984	1.00
		Direct LDL	0.557	0.987	1	0.101	0.900	0.152	0.921	0.971	0.987
		TG	0.879	0.107	0.101	1	0.342	−0.241	0.003	0.280	0.110
		TC	0.714	0.909	0.900	0.342	1	0.387	0.941	0.938	0.915
		HDL	−0.146	0.141	0.152	−0.241	0.387	1	0.498	0.094	0.156
		Anandraja LDL	0.443	0.929	0.921	0.003	0.941	0.498	1	0.897	0.934
		LDL-Chen	0.704	0.984	0.971	0.280	0.938	0.094	0.897	1	0.985
		New formula	0.571	1.00	0.987	0.110	0.915	0.156	0.934	0.985	1
	Total	Ahmadi LDL	1	0.566	0.553	0.876	0.697	−0.157	0.430	0.696	0.566
		Friedwald LDL	0.566	1	0.982	0.101	0.907	0.149	0.922	0.979	0.994
		Direct LDL	0.553	0.982	1	0.096	0.894	0.155	0.910	0.963	0.978
		TG	0.876	0.101	0.096	1	0.319	−0.262	−0.011	0.272	0.106
		TC	0.697	0.907	0.894	0.319	1	0.396	0.944	0.936	0.917
		HDL	−0.157	0.149	0.155	−0.262	0.396	1	0.509	0.094	0.159
		Anandraja LDL	0.430	0.922	0.910	0.011	0.944	0.509	1	0.893	0.931
		Chen LDL	0.696	0.979	0.963	0.272	0.936	0.094	0.893	1	0.986
		New formula	0.566	0.994	0.978	0.106	0.917	0.159	0.931	0.986	1

Sensitivity and specificity of various methods are provided in Table 6. In general, we found that Anandraja equation had the most sensitivity (89.5%), while the most specificity was for the new formula (98.9%). Considering the Yuden index, Friedwald obtained the first rank (86.2%) that is followed by the new formula (84.2%). After classification by cut-off point of 100 mg/dL for TG concentrations, it was revealed that the most amount for Yuden index was for Friedwald for both categories (TG < 100 mg/dL: 86.6%; TG > 100 mg/dL: 85%).

**Table 6:** Sensitivity and specificity of different LDL-C calculation methods

			Direct LDL				Sensitivity	Specificity	Youden index	*p*-value
			110>		110<					
			N	Percentage	N	Percentage				
Total	Ahmadi LDL	Low	2539	80.7	230	33	67	80.7	47.7	< 0.001
		High	609	19.3	466	67				
	Friedwald LDL	Low	3087	98.1	83	11.9	88.1	98.1	86.2	< 0.001
		High	61	1.9	613	88.1				
	Anandraja LDL	Low	2850	90.5	73	10.5	89.5	90.5	80	< 0.001
		High	298	9.5	623	89.5				
	Chen LDL	Low	3108	98.7	187	26.9	73.1	98.7	71.8	< 0.001
		High	40	1.3	509	73.1				
	New formula	Low	3113	98.9	102	14.7	85.3	98.9	84.2	< 0.001
		High	35	1.1	594	85.3				
TG < 100	Ahmadi LDL	Low	2269	98.1	230	49.5	50.5	98.1	48.6	< 0.001
		High	45	1.9	235	50.5				
	Friedwald LDL	Low	2273	98.2	54	11.6	88.4	98.2	86.6	< 0.001
		High	41	1.8	411	88.4				
	Anandraja LDL	Low	2076	89.7	44	9.5	90.5	89.7	80.2	< 0.001
		High	238	10.3	421	90.5				
	Chen LDL	Low	2310	99.8	155	33.3	66.7	99.8	66.5	< 0.001
		High	4	0.2	310	66.7				
	New formula	Low	2291	99	66	14.2	85.8	99	84.8	< 0.001
		High	23	1	399	85.8				
TG > 100	Ahmadi LDL	Low	270	32.4	0	0	100	32.4	32.4	< 0.001
		High	564	67.6	231	100				
	Friedwald LDL	Low	814	97.6	29	12.6	87.4	97.6	85	< 0.001
		High	20	2.4	202	87.4				
	Anandraja LDL	Low	774	92.8	29	12.6	87.4	92.8	80.2	< 0.001
		High	60	7.2	202	87.4				
	Chen LDL	Low	798	95.7	32	13.9	86.1	95.7	81.8	< 0.001
		High	36	4.3	199	86.1				
	New formula	Low	822	98.6	36	15.6	84.4	98.6	83	< 0.001
		High	12	1.4	195	84.4				

## Discussion

In the present study, we found that compare to four common formulas, Friedwald was the best equation to estimate LDL-C concentrations in Iranian children and adolescents and the new formula was the next accurate equation.

One of main identified potential risk factors for CVD and atherosclerosis in adulthood is high concentration of LDL-C. Research in children and adolescents has revealed that monitoring lipid profile status at young age can be helpful to prevent CVD at adulthood [15]. Accordingly, studding on various methods to find the most accurate one can be helpful to reduce CVD events.

To the best of our knowledge, most studies on comparing formulas to estimate LDL-C concentrations have been conducted on adult populations [6, 7, 12, 13, 16, 17]. Martin et al., examined four equations including Friedewald, Chen, de Cordova, and Hattori compare to direct measurement in hospitalized patients in South Africa. They found a favorable correlation between the de Cordova formula and Friedewald at low TG concentrations. However, the Hattori formula was the best equation to estimate LDL-C in hospitalized patients, even at extreme lipid values [13]. According to Wadhwa et al.,‘s study, among 7 formulas, Friedewald, Cordova, Vujovic, Ahmadi, Anandaraja, Puavillai and Hattori, Vujovic formula was the most accurate one in Indian adult population [18]. Krishnavena et al., also reported that Friedwald correlated maximally with direct measurement of LDL-C at all levels of TG except at TG less than 100 mg/dL in an Indian adult population. They found that for subjects with serum levels of TG < 100 mg/dl, Anandaraja’s Formula was the most accurate equation [19]. Different findings between our study and the aforementioned ones are likely to be due to differences in age range, race, and different estimation formulas.

Ahmadi et al., reported that in Iranian adult subjects with low TG concentrations and undesirably high TC, Friedewald equation may overestimate LDL-C. Therefore, they suggested a new formula for such subjects and named it as Admadi formula [20]. Although Ahmadi equation was developed based on Iranian adult populations [20], we found that it cannot be appropriate for children and adolescents and it showed the lowest correlation with direct measurement (*r* = 0.553). Accordingly, we can conclude that considering age range plays a crucial role on choosing an accurate estimation formula.

It seems only one study compared LDL-C formulas in subjects younger than 18 years old [9]. Garoufi et al., compared calculated LDL-C using Anandaraja and Friedwald formulas with directly measured LDL-C in 1005 healthy and dyslipidemic children (age range: 2–18 yrs. old) in Greece. They showed that using Friedwald formula, serum levels of LDL-C was lower than the measured value in 75.6% of healthy and in 77.3% of dyslipidemic children. They also found that Friedwald formula was more accurate screening tool compared to Anandaraja equation in healthy participants, while Anandaraja was more appropriate for following-up dyslipidemic children [9]. Our findings were in line with the mentioned study. In our study, Friedwald equation was the most accurate one. However, we did not do a classification based on LDL-C to compare healthy and dyslipidemic children. In addition, the correlation between Friedwald and direct assay was a little bit greater in our study compare to Garoufi et al’.,s study (0.98 vs. 0.97).

Although Frielwald formula has several limitations, it seems this formula is still the most accurate one compare to the other four formulas in our children and adolescent society.

Our study had several limitations. First, we did not use a reference method to measure LDL-C. Second, the comparisons were conducted only among four common formulas and we cannot make a decision regarding the accuracy of other estimation formulas. Third, we cannot clarify whether the new formula can be accurate for non-fasting measurements or not. However, the present study seems to be the first study to compare estimation LDL-C formulas among children and adolescents at national levels in Asia. We also compared estimation formulas for both lower and higher TG values. In addition, we developed and introduced a new formula with relatively similar accuracy to Friedwald on a representative sample of our children and adolescent society. In the present study, apart from correlations of equations with direct assay, sensitivity, specificity and the Yuden index for each were also reported.

## Conclusion

It is concluded that Friedwald was the best equation to estimate LDL-C concentrations in Iranian children and adolescents and the new formula was the next accurate equation. In addition, Friedwald formula was the most accurate formula to estimate LDL-C in children and adolescent with either low or high TG values.

## Data Availability

The data was obtained from the corresponding author.

## Abbreviations

LDL-C: Low density lipoprotein-Cholesterol
TG: Triglyceride
VLDL-C: Very-low-density lipoprotein cholesterol
TC: Total cholesterol
HDL-C: High-density lipoprotein-cholesterol
R^2^: Coefficient of determination
